# Cor triatriatum dexter

**DOI:** 10.1002/ccr3.1526

**Published:** 2018-04-14

**Authors:** Dominika Zoltowska, Jagadeesh K Kalavakunta

**Affiliations:** ^1^ Department of Internal Medicine Western Michigan University Homer Stryker School of Medicine 300 Portage Street Kalamazoo 49007 Michigan; ^2^ Department of Cardiology Michigan State University/Borgess Medical Center 1521 Gull Rd Kalamazoo 49048 Michigan

**Keywords:** Arrhythmia, cardiovascular disease, echocardiography, embryology

## Abstract

Cor triatriatum dexter (CTD) is an extremely rare finding (<0.01%), resulting from the persistence of the right valve of sinus venosus. Echocardiography with color Doppler is the first‐line tool for diagnosis and decision making.

## Case

**Figure 1:**
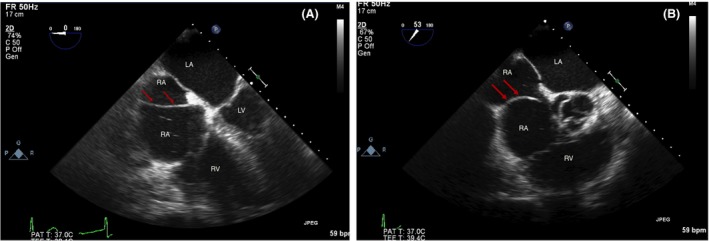
Transesophageal echocardiogram (A and B). Red arrows indicating a membrane dividing RA into two chambers. LA, left atrium; RA, right atrium; LV, left ventricle; RV, right ventricle.

A 75‐year‐old man was referred to our clinic for further treatment of atrial fibrillation. Physical exam revealed irregularly irregular rhythm and no signs of heart failure. Transthoracic echocardiogram was performed and showed preserved left ventricular ejection fraction, severely dilated left atrium, and moderately dilated right atrium (RA) with a membrane originating from the inferior vena cava orifice, extending to the atrial septum. Small interatrial communication was noted. Subsequent transesophageal echocardiogram confirmed the presence of the membrane dividing the RA into two chambers, consistent with the diagnosis of cor triatriatum dexter (CTD) (Fig. 1). Two parts of RA were communicating without flow restriction, and no significant shunt across atria was registered.

Clinical questions: What is the management of CTD?

Patients with obstructive CTD require surgical resection or percutaneous catheter disruption of the membrane 1. Our patient had mild septation without flow disturbances; thus, the strategy of watchful observation was adopted.

## Authorship

DZ and JK: were the physicians seeing the patient in the clinic. DZ and JK: were responsible for performing, diagnosing, and discussing the imaging studies of the patient. DZ: prepared the manuscript draft, which was critically revised and approved by JK.

## Conflict of Interest

None declared.

## References

[1] Moral, S. , E. Ballesteros , M. Huguet , A. Panaro , J. Palet , and A. Evangelista . 2016 Differential diagnosis and clinical implications of remnants of the right valve of the sinus venosus. J. Am. Soc. Echocardiogr. 29:183–194. 26787493 10.1016/j.echo.2015.11.018

